# Comprehensive Understanding of the Bacterial Populations and Metabolites Profile of Fermented Feed by 16S rRNA Gene Sequencing and Liquid Chromatography–Mass Spectrometry

**DOI:** 10.3390/metabo9100239

**Published:** 2019-10-21

**Authors:** Wei Jin, Zheng Zhang, Kun Zhu, Yanfeng Xue, Fei Xie, Shengyong Mao

**Affiliations:** 1 Laboratory of Gastrointestinal Microbiology, Jiangsu Key Laboratory of Gastrointestinal Nutrition and Animal Health, College of Animal Science and Technology, Nanjing Agricultural University, Nanjing 210095, China; jinwei@njau.edu.cn (W.J.); 2016105045@njau.edu.cn (Z.Z.); 2016805092@njau.edu.cn (K.Z.); xueyanfeng1990@163.com (Y.X.); 2017105050@njau.edu.cn (F.X.); 2 National Center for International Research on Animal Gut Nutrition, Nanjing Agricultural University, Nanjing 210095, China; 3 National Experimental Teaching Demonstration Center of Animal Science, Nanjing Agricultural University, Nanjing 210095, China

**Keywords:** fermented feed, bacterial population, small molecular metabolites, *Lactobacillus salivarius*, *Bacillus subtilis*, *Saccharomyces cerevisiae*

## Abstract

The comprehensive bacterial populations and metabolites profile in fermented feed is unclear, which may have significant effects on the stability of fermented feed quality and animal gut health. In this study, 16S rRNA gene sequencing and liquid chromatography–mass spectrometry were used to explore the bacterial populations and metabolites profile in the fermented feed incubated with probiotics (MF) or without probiotics (SF). The probiotics were a combination of *Lactobacillus salivarius*, *Bacillus subtilis,* and *Saccharomyces cerevisiae*. The pH and lactic acid levels were higher in MF than in SF (*P <* 0.05), while the total volatile fatty acid content was lower (*P <* 0.05). Interestingly, after fermentation, the most abundant bacterial genus in MF was *Enterococcus*, rather than the added probiotics *Lactobacillus* or *Bacillus*. *Weissella* and a few potential pathogens (*Enterobacter, Escherichia-Shigella,* and *Pantoea*) were dominant in SF (*P <* 0.05). Metabolomics analysis identified 32 different metabolites in the two types of fermented feed. These metabolites enriched in MF, such as maleic acid, phenylacetic acid, ethyl linoleate, dihomo-gamma-linolenic acid, and L-theanine had potential antimicrobial activities. Conclusively, the addition of probiotics enriched a few potentially beneficial microbes and small molecular compounds with antimicrobial activities, and inhibited the potential pathogens in fermented feed.

## 1. Introduction

Fermented feeds have been widely investigated for their potential to reduce the use of antibiotics and growth promoters in livestock and the feed costs [1,2]. The positive influence of fermented feed on animal gut health is thought to be related to the beneficial effects of probiotics, microbial metabolites, and prebiotics [3]. Wang et al. reviewed the microbiota and biochemical changes in fermented feed and identified *Lactobacillus*, *Bacillus,* and yeasts as the major microorganisms in these feeds [3]. The microbial populations in fermented feeds are thought to consist mainly of the inoculated probiotics, which inhibit the growth of endogenous pathogens, such as coliforms, *Salmonella*, and molds. However, most evaluations of fermented feed quality have, therefore, monitored only a few probiotics and pathogens [2,4,5]. Few studies have investigated the whole microbial community of the fermented feed. Consequently, the composition of the actual microbial populations in the fermented feed is unclear. Similarly, fermented feed analysis usually involves the determination of the main nutrient components, such as crude protein, crude fiber, total phosphorus, lactic acid, short chain fatty acids, small-sized peptides, amino acids, and a few anti-nutritional factors [6,7,8]. Consequently, the comprehensive profile of small molecular microbial metabolites in fermented feed is also unclear. Nevertheless, these neglected microorganisms and small molecular metabolites may have significant effects on the stability of fermented feed quality and animal gut health.

We postulate that to improve the stability of fermented feed quality and animal gut health depends on comprehensively understanding the microbial populations and metabolites in fermented feed. To facilitate this, in this study, we carried out two types of fermentation. Feed was incubated with or without the addition of probiotics (*L. salivarius*, *B. subtilis*, and *S. cerevisiae*). After fermentation, 16S rRNA gene sequencing and liquid chromatography–mass spectrometry (LC/MS) were used to investigate the bacterial populations and small molecular compounds in the two types of fermented feed.

## 2. Results

### 2.1. Lactic Acid and Volatile Fatty Acid Concentrations and pH in the Fermented Feed

As shown in Table 1, the pH and lactate concentrations were higher in MF than in SF (*P <* 0.05). The acetate, propionate, isobutyrate, and total volatile fatty acid concentrations were lower in MF than SF (*P <* 0.05). Butyrate was not detected in either group.

### 2.2. Bacterial Community Composition in the Fermented Feed

A total of 629,917 sequences remained after quality filtering, and 2475 OTUs were identified at the 97% similarity level. The average sequence number per sample was 78,740. The rarefaction curves approaching a plateau indicated that the sampling could cover the majority of the bacterial diversity (Supplementary Figure S1).

**Figure 1:**
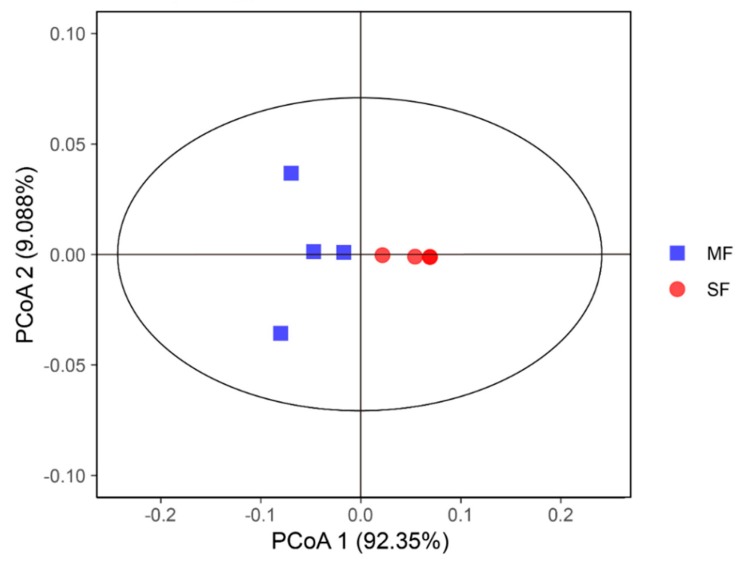
Principal coordinate analysis of microflora in fermented feed samples.

As shown in Table 2, the number of OTUs (*P =* 0.248), Chao1 (*P =* 0.564) and Shannon indexes (*P =* 0.772) showed no significant differences between MF and SF. The Ace index (*P =* 0.021) was lower and the Simpson index (*P =* 0.037) was higher in MF than SF. The PCoA analysis showed that the SF samples were separated from the MF samples (Figure 1).

**Figure 2:**
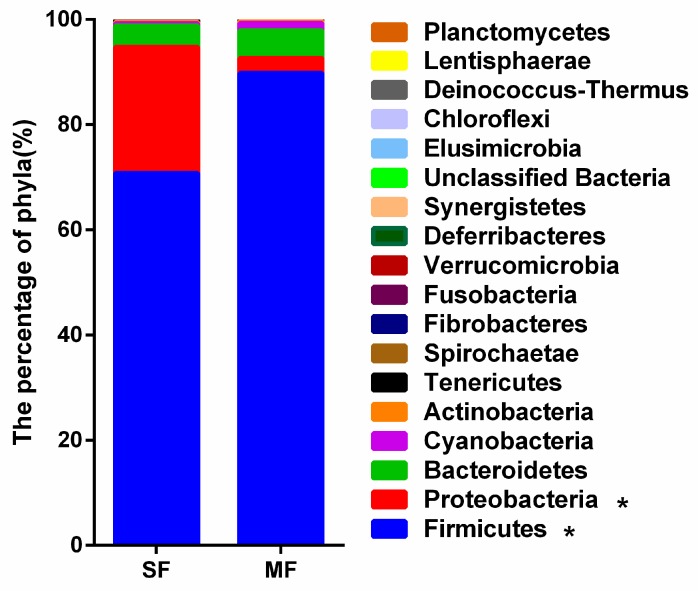
Phylum-level taxonomic composition of the bacterial communities in fermented feed samples.

At the phylum level, 18 phyla were identified (Figure 2). The three most dominant phyla were Firmicutes (80.29%), Proteobacteria (13.41%), and Bacteroidetes (4.66%). The relative abundance of Firmicutes was higher in MF than in SF (*P =* 0.021) and the relative abundance of Proteobacteria was lower in MF than in SF (*P =* 0.021).

**Figure 3:**
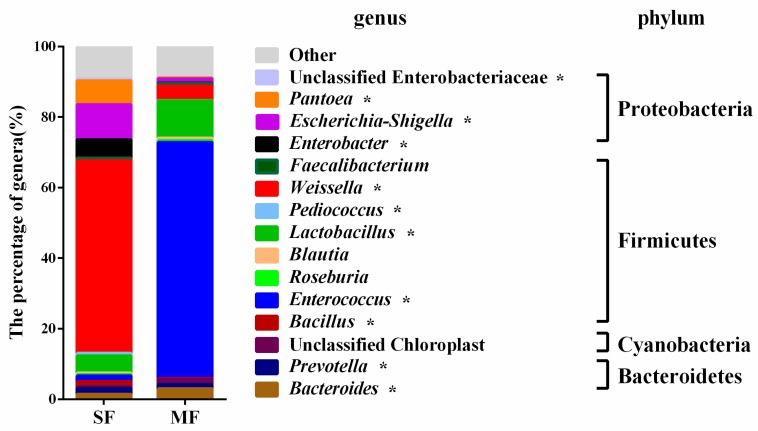
The average relative abundance at the genus level (% of total sequences) in fermented feed samples. Only genera with a relative abundance ≥ 0.5% in at least one treatment are shown.

As shown in Figure 3, 15 genera had relative abundances greater than 0.5% in at least one sample. The relative abundances of *Bacteroides* (*P =* 0.043), *Enterococcus* (*P =* 0.021), and *Lactobacillus* (*P =* 0.021) were higher in MF than in SF, while the relative abundances of *Prevotella* (*P =* 0.021), *Bacillus* (*P =* 0.021), *Pediococcus* (*P =* 0.043), *Weissella* (*P =* 0.021), *Enterobacter* (*P =* 0.021), *Escherichia-Shigella* (*P =* 0.021), *Pantoea* (*P =* 0.021), and *Unclassified Enterobacteriaceae* (*P =* 0.021) were lower in MF than in SF.

**Figure 4:**
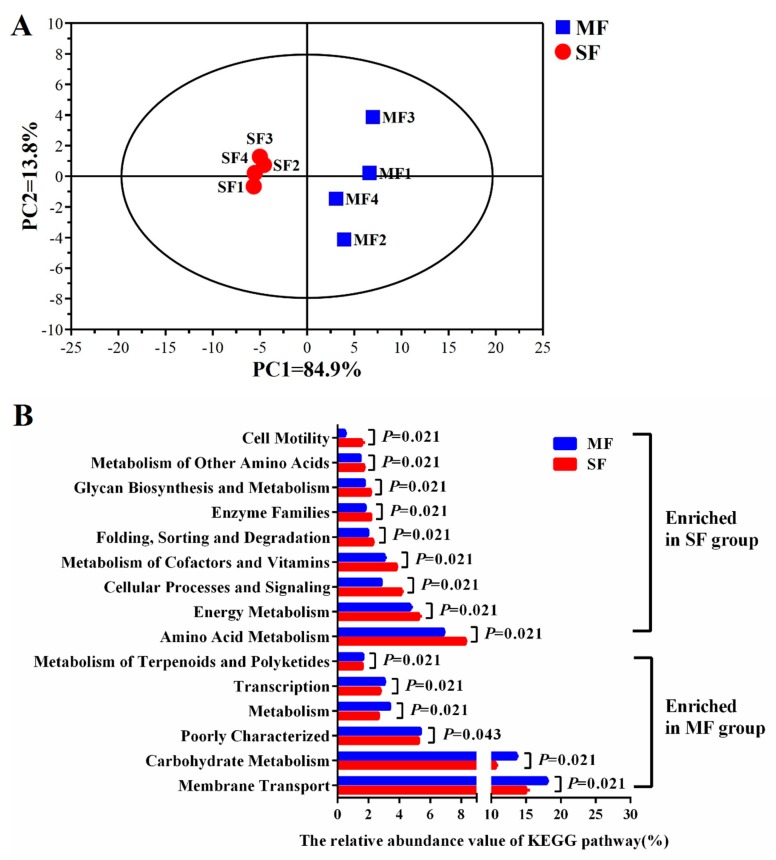
Functional predictions of microbes in fermented feed samples. (**A**) The principal component analysis (PCA) of gene pathways of microbes in fermented feed. (**B**) Effect of combined microbial fermentation on the abundance of Kyoto Encyclopedia of Genes and Genomes (KEGG) pathways (relative abundance >1% and *P* < 0.05).

The microbial functions were distinct between MF and SF (Figure 4). Genes related to the transcription, membrane transport, metabolism, carbohydrate metabolism, and terpenoid and polyketide metabolism were enriched in MF compared to SF (*P <* 0.05). Genes involved in amino acid metabolism; energy metabolism; glycan biosynthesis and metabolism; cellular processes and signaling; metabolism of cofactors and vitamins; protein folding, sorting and degradation; enzyme families; and cell motility were enriched in SF compared to MF (*P <* 0.05).

### 2.3. Small Molecular Metabolites in the Fermented Feed

**Figure 5:**
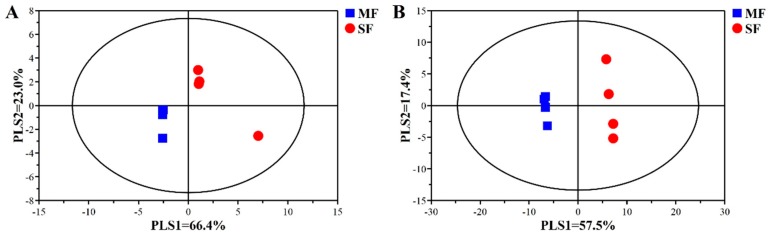
Partial least squares discriminant analysis (PLS-DA) of compounds from fermented feed samples in positive and negative mode. (**A**) PLS-DA score plot [predictive ability parameter (Q2) (cumulative) = 0.906, goodness-of-fit parameter (R2) (Y) = 0.951] in positive mode. (**B**) PLS-DA score plot [predictive ability parameter (Q2) (cumulative) = 0.994, goodness-of-fit parameter (R2) (Y) = 1] in negative mode.

In total, 109 metabolites were identified. As shown in Figure 5, the partial least squares discriminant analysis (PLS-DA) revealed an obvious and regular variation for both feed samples in both ionization modes. In the positive mode (Figure 5A), the PLS-DA revealed that PLS axes 1 and 2 accounted for 66.4% and 23.0% of the total variation, respectively. In the negative mode (Figure 5B), the PLS-DA showed that PLS axes 1 and 2 accounted for 57.5% and 17.4% of the total variation, respectively. Integrating the results of the statistical analyses and the VIP values obtained from the PLS-DA (FDR < 0.05 and VIP > 1.2) identified 32 different metabolites (Table 3), including organic acids, lipids, amino acids, and nucleosides/nucleotides. MF was enriched in 22 compounds, such as maleic acid, phenylacetic acid, ethyl linoleate, dihomo-gamma-linolenic acid, L-theanine, glutamine, glutamic acid, and 2-deoxypentose. SF was enriched in 10 metabolites, including glucopyranuronic acid, malonic acid, butyl levulinate, arginine, 2’-deoxyinosine, and slaframine.

### 2.4. Correlation between the Bacterial Populations and Small Molecular Metabolites in the Fermented Feed

**Figure 6:**
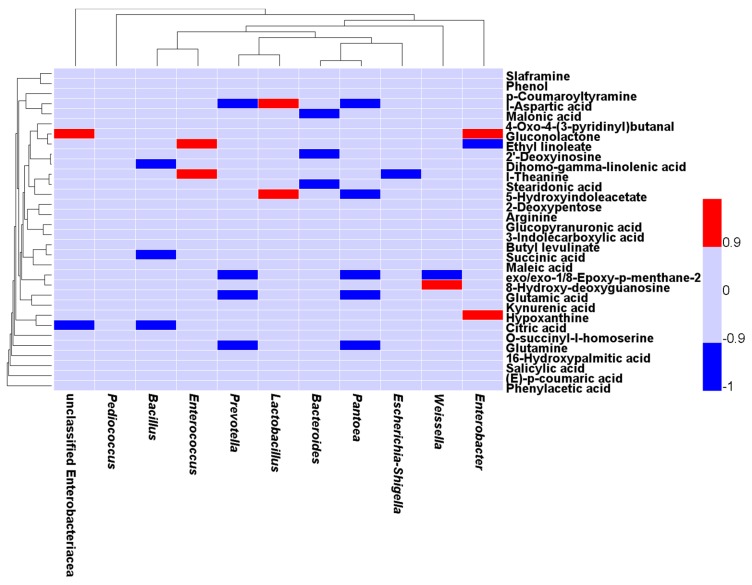
Correlations between microbes and metabolites in fermented feed samples. Only the genera (relative abundance ≥ 0.5%, *P <* 0.05) for which abundance was significantly related to compounds (VIP > 1.2) are presented. The Spearman correlation coefficient was used to assign a color to the cells. Red indicates a significantly positive correlation (r > 0.900, *P <* 0.05) and blue indicates a significantly negative correlation (r < −0.900, *P <* 0.05).

As shown in Figure 6, *Enterococcus* was positively related to ethyl linoleate (*r =* 0.905, *P =* 0.005), and L-theanine (r = 0.952, *P =* 0.001). *Lactobacillus* was positively related to 5-hydroxyindoleacetate (r = 0.976, *P <* 0.001) and L-aspartic acid (r = 0.952, *P =* 0.001). *Weissella* was positively correlated with 8-hydroxy-deoxyguanosine (r = 0.905, *P =* 0.002) and negatively correlated with exo, exo-1, 8-epoxy-p-menthane-2, 6-diol (r = −0.929, *P =* 0.002). *Escherichia-Shigella* was negatively correlated with L-theanine (r = −0.905, *P =* 0.005). *Prevotella* and *Pantoea* were negatively correlated with exo, exo-1, 8-epoxy-p-menthane-2, 6-diol, glutamine, glutamic acid, and L-aspartic acid (r < −0.90, *P <* 0.05). *Pantoea* was negatively correlated with 5-hydroxyindoleacetate (r = −0.905, *P =* 0.005). *Enterobacter* was positively correlated with hypoxanthine (r = 0.905, *P =* 0.005) and gluconolactone (r = 0.929, *P =* 0.002) and negatively correlated with ethyl linoleate (r = −0.952, *P =* 0.001) and diethyl phthalate (r = −0.952, *P =* 0.001). *Bacteroides* was negatively correlated with malonic acid (r = −0.905, *P =* 0.005), 2’-deoxyinosine (r = −0.976, *P <* 0.001), and stearidonic acid (r = −0.905, *P =* 0.005). *Bacillus* was negatively correlated with dihomo-gamma-linolenic acid (r = −0.905, *P =* 0.005), citric acid (r = −0.976, *P <* 0.001), and succinic acid (r = −0.952, *P =* 0.001). *Unclassified Enterobacteriaceae* was positively correlated with gluconolactone (r = 0.916, *P =* 0.002) and negatively correlated with citric acid (r = −0.916, *P =* 0.003).

## 3. Discussion

The preliminary data showed that lactic acid bacteria were 2.5 × 10^12^ cfu/g, *B. subtilis* was 1.5 × 10^7^ cfu/g and yeast was 1.7 × 10^7^ cfu/g in the fermented feed (MF). Based on this result, the lactic acid bacteria rather than yeast dominated in the fermented feed.

The addition of probiotics (*L. salivarius*, *B. subtilis*, and *S. cerevisiae*) greatly affected the final bacterial populations in the fermented feeds. After fermentation, different bacterial populations formed in SF and MF. Many more beneficial microbes and small molecular compounds with high antimicrobial activity were present in MF than in SF. By contrast, SF had a higher abundance of potential pathogens. The content of total volatile fatty acids was much higher in SF than MF, suggesting that many more nutrients were metabolized by the SF microbes. It was a feed nutrient loss for animals in SF, compared to MF.

*Weissella* was the most dominant genus (54.6%) in SF. *Weissella* are obligate heterofermentative lactic acid bacteria; some species of this genus can be found in salted and fermented foods (e.g., kimchi and jeotgal) and play an important role in the fermentation process [9]. The relative abundances of *Enterobacter*, *Escherichia-Shigella,* and *Pantoea* were much higher in SF than in MF. These three genera belong to the *Enterobacteriaceae* and are known potential pathogens. Their higher abundance suggested that spontaneous fermentation may be highly detrimental to animal health. However, it should be noted that the viability of these pathogens was unknown. *Enterococcus,* which includes lactic acid-producing and acid-tolerant bacteria, was the most dominant genus (66.9%) in MF [10,11]. A few species in this genus, such as *Enterococcus faecium* and *Enterococcus faecalis*, have been used as probiotics to protect against intestinal disorders and pathogens [11,12,13]. The enrichment of *Enterococcus* in MF is likely related to the addition of probiotics; however, the underlying mechanism is unclear. It might be partly due to the high level of lactic acid produced by *L. salivarius* in a short time and the low pH at the beginning of fermentation. The much lower abundance of the potential pathogens in MF could be due to the high level of lactic acid, low pH and the antimicrobial activity of the beneficial microbes and their metabolites [14,15,16]. However, it should be noted that not all species in the genus *Enterococcus* were beneficial.

MF had much higher levels of maleic acid, phenylacetic acid, ethyl linoleate, dihomo-γ-linolenic acid, 1,8-epoxy-p-menthane-2,6-diol, and L-theanine. Maleic acid has a higher antimicrobial activity under acidic conditions when compared with other organic acids [17], and it has been used as an acidity agent in the food and beverage industry. Phenylacetic acid has broad-spectrum antibiotic characteristics against both Gram-positive and gram-negative bacteria and against soil-borne phytopathogenic fungi [18,19]. Kimetal. [19] reported that phenylacetic acid could inhibit the growth of *B. subtilis*, *Lactobacillus plantarum*, *Escherichia coli*, and *Staphylococcus aureus*, but it did not affect the growth of *Enterococcus faecalis* or *Lactobacillus brevis*. In the current study, the abundance of *Bacillus* was much lower in MF than in SF and was negatively correlated with the phenylacetic acid content. The higher phenylacetic acid content may therefore partly explain the low relative abundance of *Bacillus* and the high abundance of *Enterococcus* in MF.

Ethyl linoleate and dihomo-γ-linolenic acid were significantly higher in MF than in SF. Ethyl linoleate is an ester compound formed from ethanol and linoleic acid. Yeasts can catalyze this type of esterification reaction, and the combined fermentation of lactic acid bacteria and yeast is more conducive to the formation of these esters [20]. Ethyl linoleate has antibacterial activity [21], and its abundance was negatively correlated with the *Enterobacter* pathogens. Dihomo-gamma-linolenic acid, which is synthesized from gamma-linolenic acid, is a precursor of a large family of anti-inflammatory eicosanoids [22,23]. 1,8-Epoxy-p-menthane-2,6-diol (epomediol), a terpenoid compound that reportedly stimulates bile acid synthesis [24], has been used in the symptomatic treatment of the itching due to intrahepatic cholestasis of pregnancy.

A large number of free amino acids were detected in both the SF and MF and probably reflect the degradation of protein during microbial fermentation. L-theanine, a non-protein amino acid, was found in higher abundance in MF than in SF. It has positive effects on relaxation, cognitive performance, emotional state, and sleep quality and has therapeutic benefits in the treatment of cancer, cardiovascular diseases, obesity, and the common cold [25]. The metabolism, health effects, and safety of L-theanine have been reviewed by Türközü and Şanlier [26]. L-theanine could be synthesized from glutamine and ethylamine by glutaminase [27]. Therefore, these small molecular metabolites enriched in MF may play important roles in the fermented feed quality and animal gut health.

Additionally, it should be noted that only four samples per group were used and the conclusions that might be drawn from such a small sample size were limited.

## 4. Materials and Methods

### 4.1. Microorganisms, Culture Preparation, and Feed Fermentation Progress

Three microbial strains, *Lactobacillus salivarius* CGMCC No. JCM1231 (L), *Bacillus subtilis* CGMCC No. SB3295 (B), and *Saccharomyces cerevisiae* CGMCC No. CICC1464 (S), were used to ferment the feed. The L strain was grown in Man Rogosa Sharpe (MRS) medium; the B strain was grown in beef extract-peptone-yeast extract (BPY) medium; and the S strain was grown in yeast extract-peptone-dextrose (YPD) medium; all strains were incubated at 37 °C for 12 h. The concentrations of the three strains were at 10^8^ cfu/mL in cultures. The cultures of the three strains were mixed (L:B:S = 2:2:1) to prepare the inoculum. The compound feed consisted of corn powder, soybean meal, and bran (7:2:1, w/w). The compound feed, sterile water, and inoculum were fully mixed at a ratio of 66:32:2 (w/v/v), then transferred to a plastic bag equipped with a one-way valve (Rou Duoduo Biotechnology Co., Beijing, China) and incubated at 37 °C for 48 h according to the methods of Hong et al. [28]. In the control feed (SF), the inoculum was replaced with sterilized culture medium. At the end of the fermentation, samples were collected and stored at −80 °C for subsequent determination of the bacterial composition and metabolites.

### 4.2. Determination of pH and Volatile Fatty Acid (VFA) and Lactate Contents

The pH of the fermented feeds was determined with a Hanna HI 9024C digital pH meter (Hanna Instruments, Woonsocket, RI, USA). The volatile fatty acids (VFAs) were determined by gas chromatography (Agilent 7890B, Agilent, California, USA) according to Jin et al. [29]. Lactate was determined using a lactic acid assay kit (Nanjing Jiancheng Bioengineering Institute, Nanjing, China).

### 4.3. DNA Extraction, 16S rRNA Gene Sequencing, and Data Analysis

Microbial DNA was extracted from 0.3 g fermented feed by bead-beating and phenol-chloroform extraction [30]. The quantity of DNA was determined with a NanoDrop 2000 spectrophotometer (Thermo Fisher Scientific, Madison, USA).

The V4 region of the bacterial 16S rRNA genes was amplified by the polymerase chain reaction (PCR) using the primers 515F (5’-barcode-GTG CCA GCM GCC GCG GTA A-3’) and 806R (5’-barcode-GGA CTA CHV GGG TWT CTA AT-3’). The PCR amplicons were used for paired-end sequencing on an Illumina MiSeq platform (Tianyi Health Science Research Institute Co., Ltd. Zhenjiang, China) according to the standard protocol. The raw data were submitted to the Sequence Read Archive (SRA) under accession number SRP158786.

The raw Illumina FASTQ data were handled with QIIME software version 1.9.1. After barcode read removal and quality control, high-quality sequences were obtained for analysis using a read trimming tool (Trimmomatic) with default parameters -phred33 MINLEN:75 [31]. Based on a 97% similarity level, operational taxonomic units (OTUs) were clustered through the use of UPARSE [32]. The Bayesian classifier of the Ribosomal Database Project [33] was used to perform the taxonomy assignment by contrasting the representative sequences of the OTU cluster and the Silva 11.9 database [34]. Community diversity was assessed based on the Ace, Chao1, Shannon, and Simpson indexes using mothur [35]. Unweighted UniFrac metric-based principal coordinate analysis (PCoA) was performed to compare sample distances between two groups [36]. PICRUSt analysis was used to predict the molecular functions of bacteria within individual samples [37]. Prior to the PICRUSt analysis, the 16S rRNA reads copy number was normalized [37]. The similarities among the bacterial functions were assessed by principal component analysis (PCA) using SIMCA-P software version 14.0 (Umetrics, Umea, Sweden).

### 4.4. Liquid Chromatography/Mass Spectrometry Analysis and Data Processing

Feed samples (50 mg) were prepared according to the method of Xue et al. [38]. Metabolites were analyzed using an Exactive Orbitrap mass spectrometer (MS) (Thermo Fisher Scientific, Bremen, Germany) interfaced with a heated electrospray ionization source, and operated at 35,000 mass resolution. The chromatographic separation was accomplished on a Hypergold C18 (3.0 μm, 4.6 × 100 mm) column at 40 °C with the following liquid chromatography (LC) parameters: injection volume, 4 μL; flow rate, 0.3 mL/min. The mobile phase was a mixture of (A) 0.1% formic acid in water and (B) 0.1% formic acid in acetonitrile. The gradient elution procedure is shown in Supplementary Table S1. The MS parameters were as follows: ionization mode, positive and negative; heater temperature, 300 °C; sheath gas flow rate, 45 arb; auxiliary gas flow rate, 15 arb; sweep gas flow rate, 1 arb; spray voltage, 3.0 KV (negative, 3.2 KV); capillary temperature, 350 °C; S-Lens RF Level, 60% (negative, 30%). The mass range for obtaining the data was restricted to 50–1000 m/z. The primary data were processed using Thermo Scientific SIEVE software for differential expression analysis (Thermo Fisher Scientific, Waltham, MA) and normalized and post-edited in Microsoft Excel 2010. The final data were formed as a two-dimensional data matrix, including the variables (rt_mz, retention time, and mass charge ratio), molecular weight (compMW), observations (samples), and peak intensity. The data, especially m/z, were then aligned with Kyoto Encyclopedia of Genes and Genomes (KEGG) Database (http://www.genome.jp/kegg/) and the online Human Metabolome database (http://www.hmdb.ca/) for identification of the detected metabolites.

### 4.5. Data Analysis

Statistical analyses were done with SPSS Statistics software version 20.0 (IBM Corp., Armonk, NY, USA). The Kruskal–Wallis test was used to assess the significant differences in the microbial phyla and genera between the two groups. A t-test was performed to assess the differences in pH, VFA, lactate, and metabolite levels. Statistical significance was declared at *P <* 0.05. The *P* values of the metabolomics and microbial data were corrected using a false discovery rate (FDR). The FDR-corrected *P* -values below 0.05 were considered statistically significant.

A partial least squares discriminant analysis (PLS-DA) was accomplished with SIMCA-P software. The PLS-DA was determined by the goodness-of-fit parameter (R2X) and the predictive ability parameter (Q2). Based on the PLS-DA results, the metabolites were plotted in accordance with their importance in the separation. Every compound had a value—the variable importance in the projection (VIP)—where VIP > 1.0 indicated that a metabolite was significantly different. The Spearman’s rank correlation coefficient was calculated with GraphPad Prism software version 6.00 (GraphPad Software, San Diego, CA, USA) to evaluate the metabolites and microbiota.

## 5. Conclusions

The addition of probiotics (*L. salivarius*, *B. subtilis*, and *S. cerevisiae*) increased the lactic acid level and deceased the total volatile fatty acid levels in fermented feeds, suggesting the preservation of many more nutrients. The abundances of a few potentially beneficial microbes and small molecular compounds with antimicrobial activities were enriched and may have helped to suppress the growth of pathogens. The bacterial populations and small molecular compound profiles revealed in the present study in fermented feeds treated with or without probiotics can, therefore, guide the production and application of fermented feeds in livestock.

## Figures and Tables

**Table 1:** The pH, lactate and volatile fatty acid concentration in fermented feed samples.

Item	SF	MF	*P*-value
pH	4.79 ± 0.09	4.97 ± 0.02	0.006
Lactate (μmol/g)	49.52 ± 5.02	98.71 ± 4.59	<0.001
Acetate (μmol/g)	153.66 ± 5.44	20.35 ± 3.65	<0.001
Propionate (μmol/g)	19.04 ± 4.72	4.97 ± 0.88	0.001
Isobutyrate (μmol/g)	6.50 ± 0.94	0.56 ± 0.04	<0.001
Total volatile fatty acid (μmol/g)	179.20 ± 7.34	25.88 ± 4.51	<0.001

**Table 2:** Alpha diversity measures of the bacterial communities in fermented feed samples.

Item	SF	MF	*P*-value
OTUs	1571 ± 19	1559 ± 114	0.248
Ace	1949 ± 33	1559 ± 114	0.021
Chao1	1949 ± 48	1945 ± 101	0.564
Shannon	2.72 ± 0.17	2.61 ± 0.31	0.772
Simpson	0.30 ± 0.02	0.36 ± 0.07	0.037

OTUs, operational taxonomic units.

**Table 3:** Identification of significant key metabolites in fermented feed sample. (variable importance in the projection (VIP) > 1.2, false discovery rate (FDR) < 0.05, FC > 1.5 or FC < 0.67).

Compounds	RT ^a^	Mass	VIP	FDR	FC ^b^
Organic acids					
Maleic acid	1.22	116	1.31	<0.001	11.86
Phenylacetic acid	3.63	136	1.30	0.002	11.10
Citric acid	0.88	192	1.31	<0.001	3.13
Kynurenic acid	3.56	189	1.31	<0.001	2.97
3-Indolecarboxylic acid	3.56	161	1.31	<0.001	2.90
Succinic acid	1.26	118	1.27	<0.001	2.10
5-Hydroxyindoleacetate	3.79	191	1.29	<0.001	1.99
(E)-p-coumaric acid	3.87	164	1.28	<0.001	1.98
Salicylic acid	3.51	138	1.30	<0.001	1.77
Glucopyranuronic acid	0.85	194	1.29	0.002	0.03
Malonic acid	0.85	104	1.29	<0.001	0.02
Lipids					
Ethyl linoleate	12.32	308	1.50	<0.001	19.66
Dihomo-gamma-linolenic acid	9.41	306	1.49	<0.001	11.70
16-Hydroxy hexadecanoic acid	9.38	272	1.24	<0.001	2.19
Stearidonic acid	7.05	276	1.20	0.002	0.54
Gluconolactone	0.88	178	1.25	<0.001	0.28
Butyl levulinate	5.26	172	1.29	<0.001	0.10
Amino acids and derivatives					
l-Theanine	0.86	174	1.28	<0.001	8.57
Glutamine	0.83	146	1.30	<0.001	3.50
O-succinyl-l-homoserine	1.25	219	1.28	<0.001	2.31
Glutamic acid	0.84	147	1.22	0.002	2.10
l-Aspartic acid	0.84	133	1.21	0.002	1.74
Arginine	0.79	174	1.30	0.002	0.03
Nucleosides, Nucleotides					
2-Deoxypentose	1.21	134	1.29	<0.001	4.86
2’-Deoxyinosine	1.18	252	1.27	<0.001	0.45
Hypoxanthine	1.18	136	1.28	<0.001	0.42
8-Hydroxy-deoxyguanosine	1.18	283	1.23	0.002	0.29
Others					
exo,exo-1,8-Epoxy-p-menthane-2,6-diol	7.95	186	1.30	<0.001	11.72
4-Oxo-4-(3-pyridinyl)butanal	3.56	163	1.31	<0.001	2.88
p-Coumaroyltyramine	4.20	283	1.22	0.002	2.07
Phenol	3.51	94	1.30	<0.001	1.79
Slaframine	3.74	198	1.31	<0.001	0.16

^a^ RT represents retention time. ^b^ FC represents fold change, the ratio of mean value of peak area obtained from the MF group and the mean value of peak area obtained from the SF group. If FC > 1, means that this metabolite is enriched in the MF group.

## Supplementary Materials

The following are available online at https://www.mdpi.com/2218-1989/9/10/239/s1: Figure S1: Rarefaction curves based on OTUs in fermented feed samples. Table S1: The procedure of gradient elution.

## References

[1] Plumed-Ferrer C., von Wright A. (2009). Fermented pig liquid feed: Nutritional, safety and regulatory aspects. J. Appl. Microbiol..

[2] Wang J., Han Y., Zhao J.Z., Zhou Z.J., Fan H. (2017). Consuming fermented distillers’ dried grains with solubles (DDGS) feed reveals a shift in the faecal microbiota of growing and fattening pigs using 454 pyrosequencing. J. Integr. Agric..

[3] Wang C., Shi C., Zhang Y., Song D., Lu Z., Wang Y. (2018). Microbiota in fermented feed and swine gut. Appl. Microbiol. Biotechnol..

[4] Missotten J.A.M., Michiels J., Goris J., Herman L., Heyndrickx M., De Smet S., Dierick N.A. (2007). Screening of two probiotic products for use in fermented liquid feed. Livest. Sci..

[5] Song D., Wang F., Lu Z., Wang Y. (2017). Effects of supplementing sow diets with *Saccharomyces cerevisiae* refermented sorghum dried distiller’s grains with solubles from late gestation to weaning on the performance of sows and progeny. J. Anim. Sci..

[6] Shi C., Zhang Y., Yin Y., Wang C., Lu Z., Wang F., Feng J., Wang Y. (2017). Amino acid and phosphorus digestibility of fermented corn-soybean meal mixed feed with *Bacillus subtilis* and *Enterococcus faecium* fed to pigs. J. Anim. Sci..

[7] Zheng L., Li D., Li Z.L., Kang L.N., Jiang Y.Y., Liu X.Y., Chi Y.P., Li Y.Q., Wang J.H. (2017). Effects of *Bacillus* fermentation on the protein microstructure and anti-nutritional factors of soybean meal. Lett. Appl. Microbiol..

[8] Zhu J., Gao M., Zhang R., Sun Z., Wang C., Yang F., Huang T., Qu S., Zhao L., Li Y. (2017). Effects of soybean meal fermented by *L. plantarum*, *B. subtilis* and *S. cerevisieae* on growth, immune function and intestinal morphology in weaned piglets. Microb. Cell Factories.

[9] Kim E., Cho Y., Lee Y., Han S.K., Kim C.G., Choo D.W., Kim Y.R., Kim H.Y. (2017). A proteomic approach for rapid identification of Weissella species isolated from Korean fermented foods on MALDI-TOF MS supplemented with an in-house database. Int. J. Food Microbiol..

[10] Ohashi Y., Ushida K. (2009). Health-beneficial effects of probiotics: Its mode of action. Anim. Sci. J..

[11] Hu Y., Dun Y., Li S., Zhang D., Peng N., Zhao S., Liang Y. (2015). Dietary Enterococcus faecalis LAB31 improves growth performance, reduces diarrhea, and increases fecal Lactobacillus number of weaned piglets. PLoS ONE.

[12] Bednorz C., Guenther S., Oelgeschläger K., Kinnemann B., Pieper R., Hartmann S., Tedin K., Semmler T., Neumann K., Schierack P. (2013). Feeding the probiotic *Enterococcus faecium* strain NCIMB 10415 to piglets specifically reduces the number of *Escherichia coli* pathotypes that adhere to the gut mucosa. Appl. Environ. Microbiol..

[13] Hu C., Xing W., Liu X., Zhang X., Li K., Liu J., Deng B., Deng J., Li Y., Tan C. (2019). Effects of dietary supplementation of probiotic *Enterococcus faecium* on growth performance and gut microbiota in weaned piglets. AMB Express.

[14] Hatoum R., Labrie S., Fliss I. (2012). Antimicrobial and probiotic properties of yeasts: From fundamental to novel applications. Front. Microbiol..

[15] Yang J.J., Niu C.C., Guo X.H. (2015). Mixed culture models for predicting intestinal microbial interactions between *Escherichia coli* and *Lactobacillus* in the presence of probiotic *Bacillus subtilis*. Benef. Microbes.

[16] Liu P., Zhao J.B., Guo P.T., Lu W.Q., Geng Z.Y., Levesque C.L., Johnston L.J., Wang C.L., Liu L., Zhang J. (2017). Dietary corn bran fermented by *Bacillus subtilis* MA139 decreased gut cellulolytic bacteria and microbiota diversity in Finishing Pigs. Front. Cell Infect. Microbiol..

[17] Paudyal R., Barnes R.H., Karatzas K.A.G. (2018). A novel approach in acidic disinfection through inhibition of acid resistance mechanisms; Maleic acid-mediated inhibition of glutamate decarboxylase activity enhances acid sensitivity of *Listeria monocytogenes*. Food Microbiol..

[18] Hwang B.K., Lim S.W., Kim B.S., Lee J.Y., Moon S.S. (2001). Isolation and in vivo and in vitro antifungal activity of phenylacetic acid and sodium phenylacetate from *Streptomyces humidus*. Appl. Environ. Microbiol..

[19] Kim Y., Cho J.Y., Kuk J.H., Moon J.H., Cho J.I., Kim Y.C., Park K.H. (2004). Identification and antimicrobial activity of phenylacetic acid produced by *Bacillus licheniformis* isolated from fermented soybean, Chungkook-Jang. Curr. Microbiol..

[20] Annan N.T., Poll L., Sefa-Dedeh S., Plahar W.A., Jakobsen M. (2003). Volatile compounds produced by *Lactobacillus fermentum*, *Saccharomyces cerevisiae* and *Candida krusei* in single starter culture fermentations of Ghanaian maize dough. J. Appl. Microbiol..

[21] Jelenko C., Wheeler M.L., Anderson A.P., Callaway B.D., McKinley J.C. (1975). Studies in burns: XIV, Heling in burn wounds treated with Ethyl Linoleate alone or in combination with selected topical antibacterial agents. Ann. Surg..

[22] Yazawa H., Iwahashi H., Kamisaka Y., Kimura K., Aki T., Ono K., Uemura H. (2007). Heterologous production of dihomo-γ-linolenic acid in *Saccharomyces cerevisiae*. Appl. Environ. Microbiol..

[23] Khan M.A.K., Yang J., Hussain S.A., Zhang H., Liang L., Garre V., Song Y. (2019). Construction of DGLA producing cell factory by genetic modification of *Mucor circinelloides*. Microb. Cell Factories.

[24] Cuevas M.J., Mauriz J.L., Almar M., Collado P.S., González-Gallego J. (2001). Effect of epomediol on ethinyloestradiol-induced changes in bile acid and cholesterol metabolism in rats. Clin. Exp. Pharmacol. Physiol..

[25] Vuong Q.V., Bowyer M.C., Roach P.D. (2019). L-theanine: Properties, synthesis, and isolation from tea. J. Sci. Food Agric..

[26] Türközü D., Şanlier N. (2017). L-theanine, unique amino acid of tea, and its metabolism, health effects, and safety. Crit. Rev. Food Sci. Nutr..

[27] Juneja L.R., Chu D.C., Okubo T., Nagato Y., Yokogoshi H. (1999). L-theanine: A unique amino acid of green tea and its relaxation effect in humans. Trend Food Sci. Technol..

[28] Hong K., Lee C., Kim S.W. (2004). *Aspergillus oryzae* GB-107 fermentation improves nutritional quality of food soybeans and feed soybean meal. J. Med. Food.

[29] Jin W., Li Y., Cheng Y., Mao S., Zhu W. (2018). The bacterial and archaeal community structures and methanogenic potential of the cecal microbiota of goats fed with hay and high-grain diets. Antonie van Leeuwenhoek.

[30] Sun Y.Z., Mao S.Y., Yao W., Zhu W.Y. (2008). DGGE and 16S rDNA analysis reveals a highly diverse and rapidly colonising bacterial community on different substrates in the rumen of goats. Animal.

[31] Caporaso J.G., Kuczynski J., Stombaugh J., Bittinger K., Bushman F.D., Costello E.K., Fierer N., Pena A.G., Goodrich J.K., Gordon J.I. (2010). QIIME allows analysis of high-throughput community sequencing data. Nat. Methods..

[32] Ye H.M., Liu J.H., Feng P.F., Zhu W.Y., Mao S.Y. (2016). Grain-rich diets altered the colonic fermentation and mucosa-associated bacterial communities and induced mucosal injuries in goats. Sci. Rep..

[33] Wang Q., Garrity G.M., Tiedje J.M., Cole J.R. (2007). Naive Bayesian classifier for rapid assignment of rRNA sequences into the new bacterial taxonomy. Appl. Environ. Microbiol..

[34] DeSantis T.Z., Hugenholtz P., Larsen N., Rojas M., Brodie E.L., Keller K., Huber T., Dalevi D., Hu P., Andersen G.L. (2006). Greengenes, a chimera-checked 16S rRNA gene database and workbench compatible with ARB. Appl. Environ. Microbiol..

[35] Schloss P.D., Westcott S.L., Ryabin T., Hall J.R., Hartmann M., Hollister E.B., Lesniewski R.A., Oakley B.B., Parks D.H., Robinson C.J. (2009). Introducing mothur: Open-source, platform-independent, community-supported software for describing and comparing microbial communities. Appl. Environ. Microbiol..

[36] Lozupone C., Knight R. (2005). UniFrac: A new phylogenetic method for comparing microbial communities. Appl. Environ. Microbiol..

[37] Langille M.G.I., Zaneveld J., Caporaso J.G., Mcdonald D., Dan K., Reyes J.A., Clemente J.C., Burkepile D.E., Thurber R.L.V., Knight R. (2013). Predictive functional profiling of microbial communities using 16S rRNA marker gene sequences. Nat. Biotechnol..

[38] Xue Y., Guo C., Hu F., Liu J., Mao S. (2018). Hepatic Metabolic Profile Reveals the Adaptive Mechanisms of Ewes to Severe Undernutrition during Late Gestation. Metabolites.

