# Supplementary material for: Comprehensive Understanding of the Bacterial Populations and Metabolites Profile of Fermented Feed by 16S rRNA Gene Sequencing and Liquid Chromatography–Mass Spectrometry
Source: Metabolites. 2019 Oct 21;9(10):239. doi: 10.3390/metabo9100239 (PMC6835224; doi:10.3390/metabo9100239)

Supporting Information

Table S1. The procedure of gradient elution.

Time(min)	Flow rate(mL/min)	A(%)	B(%)
0	0.3	95	5
2	0.3	95	5
12	0.3	5	95
15	0.3	5	95
17	0.3	95	5

Figure S1. Rarefaction curves based on OTUs in fermented feed samples.

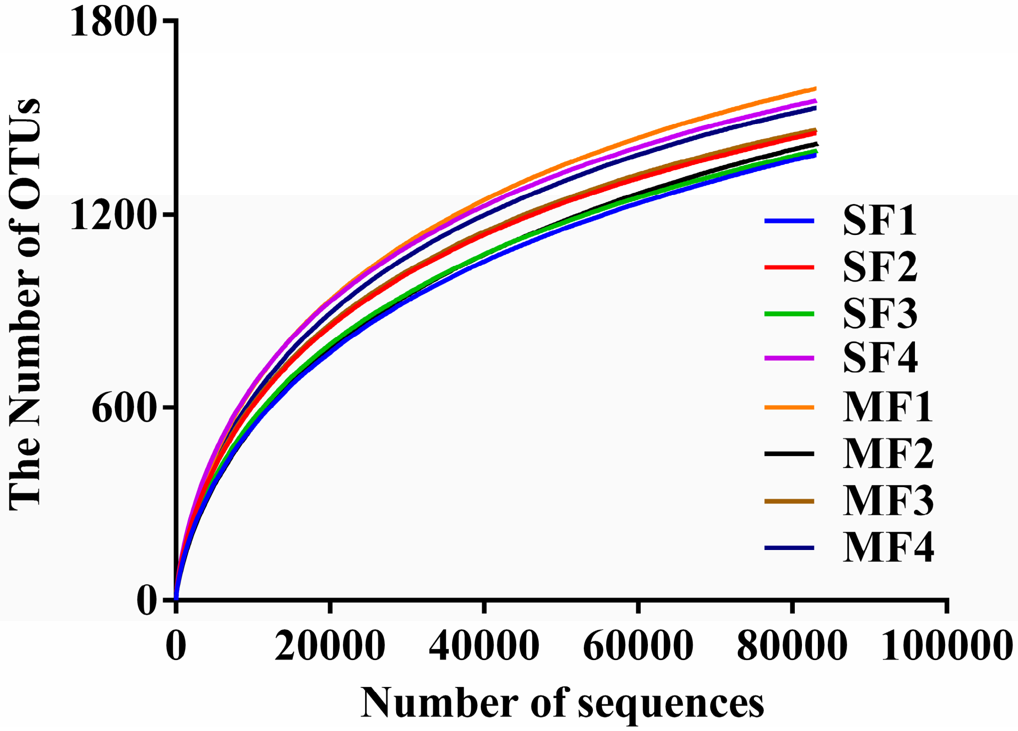

Supplement: Supplementary file 1 [file metabolites-09-00239-s001.pdf]
